# Factors associated with staff-to-resident abuse in Norwegian nursing homes: a cross-sectional exploratory study

**DOI:** 10.1186/s12913-021-06227-4

**Published:** 2021-03-19

**Authors:** Anja Botngård, Arne Henning Eide, Laura Mosqueda, Lene Blekken, Wenche Malmedal

**Affiliations:** 1 Department of Public Health and Nursing, Norwegian University of Science and Technology, Trondheim, Norway; 2 Department of Health Research, SINTEF Digital, Oslo, Norway; 3 Department of Family Medicine, Keck School of Medicine of the University of Southern California, Los Angeles, USA

**Keywords:** Risk factors, Predictors, Elder abuse, Staff-to-resident abuse, Nursing homes, Long-term care settings, Institutional care settings

## Abstract

**Background:**

Elder abuse is a public health problem that is gaining attention due to its serious impacts on people’s health and well-being, and it is predicted to increase along with the world’s rapidly ageing population. Staff-to-resident abuse in nursing homes is a complex and multifaceted phenomenon associated with multiple factors on different levels of the ecological model. This study aimed to explore individual, relational, and institutional characteristics associated with perpetrated staff-to-resident abuse in nursing homes, using a multilevel hierarchical approach.

**Methods:**

This was a cross-sectional exploratory study of 3693 nursing staff (response rate 60.1%) in 100 randomly selected nursing homes in Norway. We explored the characteristics of nursing staff, their relationship with residents, and institutional features associated with three types of abuse: psychological abuse, physical abuse, and neglect. These were modelled using multilevel mixed-effects logistic regression analyses.

**Results:**

Individual staff factors found to be associated with all three types of abuse were 1) being a registered nurse/social educator (OR 1.77–2.49) or licensed practical nurse (OR 1.64–1.92), 2) reporting symptoms of psychological distress (OR 1.44–1.46), 3) intention to leave the job (OR 1.35–1.40), and 4) reporting poor attitudes towards people with dementia (OR 1.02–1.15). Also, staff who reported poorer quality of childhood were more likely to perpetrate neglect (OR 1.14). Relational factors such as care-related conflicts (OR 1.97–2.33) and resident aggression (OR 1.36–2.09) were associated with all three types of abuse. Of institutional factors, lack of support from a manager was associated with perpetrating psychological abuse (OR 1.56).

**Conclusions:**

We found several predictors of staff-to-resident abuse on different levels of the ecological model, which underlines the importance of using a multifaceted approach to identify risk factors of elder abuse in nursing homes. However, future studies should explore the underlying mechanism and causes with a prospective or qualitative design and target the multifaceted nature of risk factors when designing preventive interventions.

## Background

Elder abuse is a public health problem affecting one out of six community-dwelling older adults worldwide [1, 2]. In nursing homes, residents are particularly vulnerable due to physical and cognitive impairments, and recent studies have found that two out of three nursing home staff admit to perpetrating abusive acts towards residents [3, 4]. Elder abuse may adversely affect a person’s physical and mental health and cause short- or long-lasting disabilities, bodily pain, somatic problems, anxiety, depression, stress, sleeping difficulties, and/or suicidal ideation, and it may increase the risk of hospitalizations, institutionalizations, and premature death [5]. Furthermore, elder abuse is related to other consequences including economic expenses and burdens by increased use of healthcare services, and those incurred by the law enforcement and criminal justice systems [5, 6].

Most research on elder abuse has been conducted in the community rather than in institutional care settings [7], even though older adults who live in institutional care settings have much significant vulnerability to abuse. Also, most studies of elder abuse have been conducted in the United States (U.S.) [8]. Previous literature has used a wide range of conceptual and operational definitions, theoretical approaches, study designs, data collection methods, and measurement instruments to capture the extent and nature of elder abuse [9–11]. The U.S. Centers for Disease Control and Prevention defines elder abuse as ‘an intentional act or failure to act by a caregiver or another person in a relationship involving an expectation of trust that causes or creates a risk of harm to an older adult’; this includes psychological, physical, sexual, financial/material abuse, and intentional or unintentional neglect [12].

**Fig. 1:**
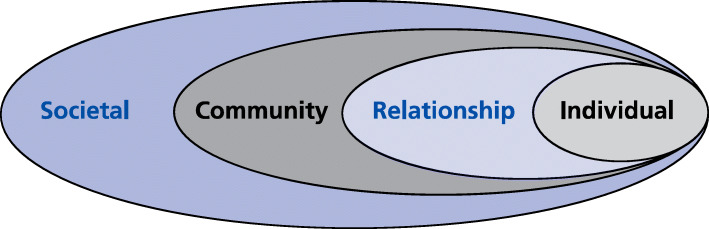
The WHO’s ecological model for understanding violence [17]

Elder abuse is a complex and multifaceted phenomenon [13] and identifying potential risk factors for staff-to-resident abuse in nursing homes is an essential first step to prevent or mitigate the mistreatment of vulnerable residents [14]. Several theories have been applied from the fields of child maltreatment, intimate partner violence, psychology, and sociology, to explain and predict causes of elder abuse [15]. However, no single theory may fully explain its nature. To accommodate its complexity, an ecological model has been recognized as valid and suitable to identify potential risk factors of elder abuse [14, 16–19]. Ecological theories of elder mistreatment have depended upon Bronfenbrenner’s *ecological model* that proposes that individuals are embedded in different environmental systems that interact with each other and the individual, and researchers have used different variations of this model as the foundation of elder abuse research [19]. The World Health Organization (WHO) outlines a four-level ecological model (Fig. 1) that illustrates the dynamic interaction and complex interplay between individual, relational, community, and societal factors, where the overlapping circles illustrate how factors at one level influence factors at the other levels [17]. The first level in this ecological model seeks to explore individual risk factors related to both the victim (resident) and the perpetrator (staff), and the second level examines their dynamic relationship, as well as their relations with other people in the immediate environment (e.g. relatives) [14]. The third level explores community contexts or institutional care factors that may influence the risk of elder abuse, and the fourth level examines the larger societal issues such as ageism, cultural norms and beliefs, and economic and social factors [14].

Previous literature has consistently reported some important factors associated with staff-to-resident abuse. Nursing home residents with physical disabilities, dementia and/or cognitive decline, high care needs, and challenging behaviours are more likely to be abused [20]. Staff characteristics that predict abuse include poor overall health, burnout or emotional exhaustion, job dissatisfaction, intention to leave the job, and holding negative attitudes towards older people [20–24]. Within families, childhood abuse has been reported as a risk factor for perpetrating elder abuse in later life [25], but to our knowledge, this has not been explored in the context of a formal caregiver/resident relationship. There are no clear demographic patterns related to staff who are abusive: studies report both young [26, 27] and older perpetrators [22], males [4, 28] and females [4], with lower [29, 30] and higher levels of education [4, 22]. People with a dementing illness often develop neuropsychiatric symptoms (NPS) such as agitation and aggressive behaviours which then relate to caregiver distress [31]. Numerous studies have posited an association between high levels of staff/resident conflicts (verbal and physical) with a higher occurrence of staff-to-resident abuse [21–23, 32, 33].

Elder abuse that occurs in institutional care is sometimes denoted as ‘institutional maltreatment’, and several individual staff characteristics may be linked to or caused by the institutional context [14]. Institutional factors such as high workload/stress, lack of social interactions or support from managers and/or co-workers, and insufficient teamwork and safety climates have been shown to influence the risk of staff-to-resident abuse [20, 23, 24, 28, 29, 34]. Moreover, facility characteristics such as size and geography have been related to the prevalence of staff-to-resident abuse [4, 21, 22, 30, 35].

The WHO (2014) emphasizes that a successful response to prevent and manage all types of violence involves a four-step public health approach that determines: (step one) the scope and consequences, (step two) causes and predictors, (step three) design, implementation, and evaluation of interventions, and (step four) evidence-based actions to monitor impact and cost-effectiveness [36]. In past decades, progress has been made in defining the extent and nature of staff-to-resident abuse in nursing homes, but research on many aspects, including the evidence of causes and predictors (step two), is still limited [2, 20]. The primary objective of this study was to explore various individual, relational, and institutional factors associated with staff-to-resident psychological abuse, physical abuse, and neglect in Norwegian nursing homes.

## Methods

### Study design

This was a cross-sectional exploratory study of nursing home staff in Norway, where the nursing homes were randomly selected from the Central Register of Establishments and Enterprises. Collection of the data was completed between October 2018 and January 2019, and it was part of a larger study aimed to measure the extent and nature, and explore the risk factors of relative-to-resident abuse, resident-to-resident aggression [37], and observed/perpetrated staff-to-resident abuse [4]. We used the STROBE guidelines for cross-sectional studies for reporting [38].

### Setting

All public and private nursing homes were eligible for inclusion. Norwegian municipalities own and run most nursing homes (> 90%), which contain both short- and long-term care units, intended for people who need a high level of care and assistance in daily activities [39]. In Norway, approximately 80% of nursing home residents have a dementing illness [40].

### Sample size and randomisation

There exist few national studies, and all studies measuring the prevalence of staff-to-resident abuse use different measurement instruments [41]. We did not statistically compute a sample size but decided to include 100 institutions, which is about 10% of all nursing homes. In comparison, the national study on elder abuse in Irish nursing homes comprised 64 out of 613 institutions [21]. To obtain a representative sample, a computerized random number generator was used to draw the 100 nursing homes. We also randomly selected 50 institutions as replacements if nursing homes declined to participate.

### Participants

Nursing staff who provided direct patient care during 3 weeks of data collection were eligible as participants. We included nursing staff working on all shifts; social educators, registered nurses, licensed practical nurses, and nursing assistants with no formal health education. In Norwegian nursing homes, an average of 31% of nursing staff are registered nurses, 2.5% are social educators, 42.5% are licensed practical nurses, and 24% are nursing assistants [42]. In Norway, registered nurses and social educators finish a bachelor’s degree, and licensed practical nurses obtain a certificate upon completion of vocational training in high school [39].

### Recruitment of nursing homes and nursing staff

The procedure of recruiting nursing homes and nursing staff is described in Botngård et al. (2020) [4]. Of the initially invited institutions, 27 declined participation, where many nursing homes were larger than the median size of 34 beds in Norway [43]. To avoid additional skewness in the sample, we started recruiting the largest institutions from the replacement list. In total, 6337 nursing staff were eligible for participating in the study, where 3811 returned their survey questionnaire (response rate of 60.1%). Some participants (*n* = 118) were excluded, mainly because they were not working in the care of nursing home residents. Overall, 3693 nursing staff participated, providing an analytical response rate of 58.3%. A flowchart of the enrolment is provided in Botngård et al. (2020) [4].

### Study variables

**Fig. 2:**
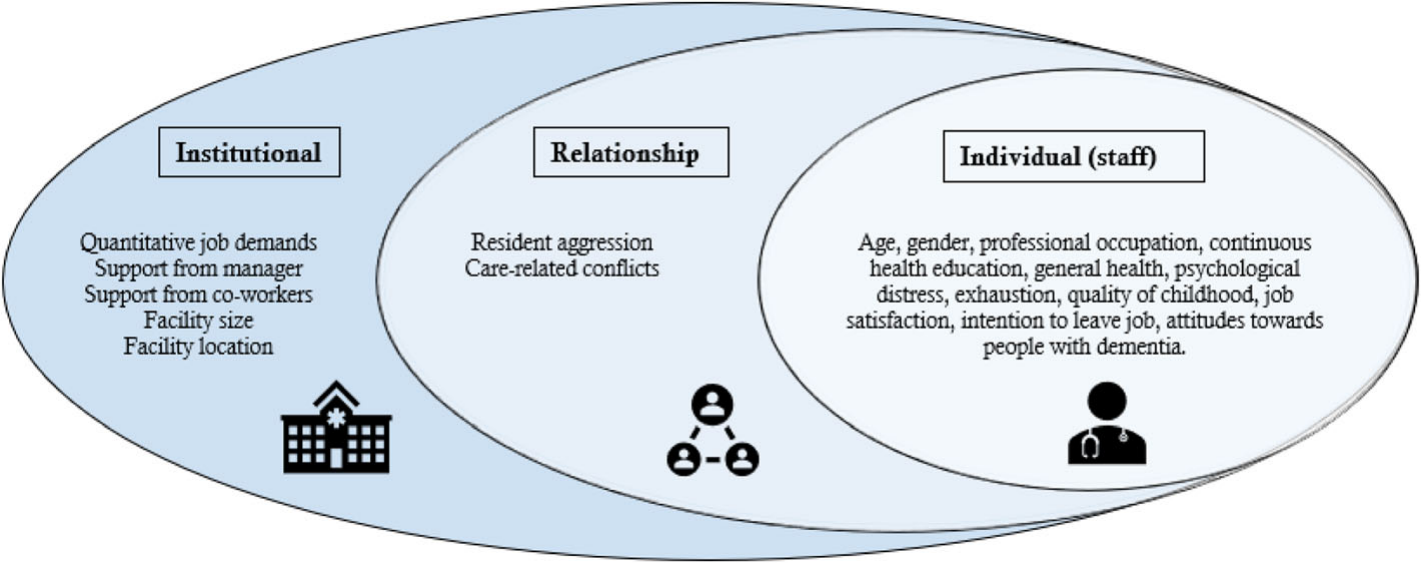
Factors (independent variables) on three out of four levels of the ecological model

The survey questionnaire used was specifically developed for this study and included different measurement instruments for the dependent and independent variables. Table 1 comprises a detailed description of the independent variables as well as the measurement instruments with Cronbach’s alpha coefficients reported in the (original) validation studies and the current study. The dependent variable was the prevalence of perpetrated psychological abuse, physical abuse, and neglect during the past year. The prevalence rates and full description of how these were measured are thoroughly described in our article on staff-to-resident abuse in Norwegian nursing homes [4]. We did not analyse sexual and financial/material abuse due to the low prevalence rates. We used WHO’s four-level ecological model and previous literature on staff-to-resident abuse to guide our choice of factors (independent variables) to include, and we explored individual factors of staff, staff/resident relational factors, and institutional factors (Fig. 2).

**Table 1:** A detailed description of the survey questionnaire and Cronbach’s alpha coefficients

Variables		Measurements	Scoring values, used in analyses	α (original study)	α (current study)
**Individual (staff)**	Sex		0 = Female 1 = Male	–	–
	Age	Years	The continuous variable used in analyses	–	–
	Occupation	Professional occupation	0 = Nursing assistant (no health education) 1 = Licensed practical nurse 2 = Registered nurse/social educator	–	–
	Continuous health education	‘Do you have continuous education in healthcare?’	0 = Yes 1 = No	–	–
	Overall health	‘How is your health in general?’	Likert scale 1–5: very good ➔ very bad	–	–
	Exhaustion	HUNT (one item) ‘Do you feel exhausted/tired?’	0 = No 1 = Yes	–	–
	Psychological distress	SCL (5 items) ‘Have you been bothered by any of these the past 14 days?’ E.g. feeling hopeless about the future, worrying too much about things.	Likert scale 1–4: not bothered ➔ very bothered Mean score cut-off ≥2.0 0 = No psychological distress 1 = Psychological distress (≥ 2.0)	0.88	0.86
	Quality of childhood	HUNT (one item) ‘When you think about your childhood, would you describe it as …’	Likert scale 1–5: very good ➔ very difficult	–	–
	Job satisfaction	‘How satisfied are you with your job in general?’	Likert scale 1–5: very satisfied ➔ very unsatisfied	–	–
	Intention to leave	‘During the past 12 months, have you considered leaving your job?’	0 = No 1 = Yes	–	–
	Attitudes	ADQ – ‘Hope’ dimension (8 items) ‘Please indicate to what extent you agree or disagree with the following statements related to dementia…’ E.g., it is important with strict routines, people with dementia are very much like children, there is no hope for people with dementia.	Likert scale 1–5: strongly agree ➔ strongly disagree Composite score 8–40 ➔ higher score = more positive ^a^	0.76	0.74
**Relational**	Resident aggression	Malmedal (5 items) ‘How often during the past 12 months have residents…’ E.g., thrown objects at you, spat at you, pinched, beat or pulled your hair.	Likert scale 1–5: daily ➔ never Average score ➔ higher score = less aggression ^a^	0.79	0.81
	Care-related conflicts	Malmedal (4 items) ‘How often during the past 12 months have it occurred conflicts between residents and staff…’ E.g., because residents refuse to eat, bathe, dress, or go to the toilet.	Likert scale 1–5: daily ➔ never Average score ➔ higher score = less conflicts ^a^	0.77	0.87
**Institutional**	Quantitative job demands	QPS_Nordic_ (4 items) ‘Is your workload irregular so that the work piles up … do you have to work overtime … is it necessary to work at a rapid pace … do you have too much to do?’	Likert scale 1–5: very seldom/never ➔ very often/always Average score ➔ higher score = more demands	0.73	0.72
	Support from manager	QPS_Nordic_ (3 items) ‘If needed, can you get support and help... are immediate superior willing to listen… are your work achievements appreciated by your immediate superior?’	Likert scale 1–5: very seldom/never ➔ very often/always Average score ➔ higher score = more support ^a^	0.83	0.85
	Support from co-workers	QPS_Nordic_ (2 items) ‘If needed, can you get support and help with your work from your co-workers … are your co-workers willing to listen to your work-related problems?’	Likert scale 1–5: very seldom/never ➔ very often/always Average score ➔ higher score = more support ^a^	0.80	0.73
	Facility size	Number of beds	The continuous variable used in analyses	–	–
	Location of municipalities	Centrality index from 1 to 6	0 = Urban (Levels 1–2) 1 = Suburban (Levels 3–4) 2 = Rural (Levels 5–6)	–	–

^a^scale/score reversed in regression analysis

### Measurements

#### Individual staff factors

Nursing staff’s overall health was measured with a single item generally accepted as useful to assess a person’s health status [44]. Psychological distress was measured with the Hopkins Symptom Checklist (SCL), an instrument widely used to measure self-reported general symptoms of anxiety and depression in population surveys, and the instrument exists in several versions with items ranging from 5 to 90 [45]. Strand et al. (2003) [45] translated the instrument into Norwegian and in the validation process, they found that the short version with only five items (SCL-5) was equally good to measure psychological distress as the versions comprising 25 items. SCL-5 measures different symptoms during the last 14 days on a 4-point Likert-scale ranging from not bothered to very bothered, and according to Strand et al. (2003) [45], a mean cut-off value of ≥2.0 qualifies as psychological distress. In the study by Strand et al. (2003) [45], Cronbach’s alpha vas reported being 0.88. When used in the current study, Cronbach’s alpha was 0.86. These same five items have also been used to measure psychological distress in a large population-based cohort in Norway, the Nord-Trøndelag Health Study (HUNT) [46]. Feelings of exhaustion and overall quality of own childhood were measured with single items previously used in HUNT [46]. Job satisfaction was measured with a single item previously found acceptable to measure the overall job satisfaction [47]. Staff’s intention to leave their jobs was measured with a single item used in other studies of elder abuse in nursing homes [21, 27].

To measure nursing staff’s attitudes towards residents with dementia, we used one subscale (‘Hope’) of the instrument, Approaches to Dementia Questionnaire (ADQ), that has been used on healthcare staff in different settings including nursing homes [48–51]. ADQ was developed by Lintern (2001) [52] as a self-report instrument to measure healthcare professionals’ attitudes towards persons with dementia, and the instrument consists of two subscales: ‘Hope’ (8 items) and ‘Recognition of Personhood’ (11 items). ‘Hope’ reflects respondents’ feelings of optimism or pessimism of the current and future condition of persons with dementia and comprises solely negatively loaded items on a 5-point Likert scale ranging from ‘strongly agree’ to strongly ‘disagree’ [52]. A composite score is obtained by summing the score of each item in the subscale (ranging from 8 to 40), where a higher score reflects more positive attitudes towards persons with dementia. This instrument was translated into Norwegian by Kada et al. (2009) [53] and used to explore the attitudes to dementia perceived by 291 nursing staff in 14 nursing homes and one hospital-based geriatric ward in Norway. However, the authors did not report any psychometric properties of the translated version. When developed by Lintern (2001) [52], the hope dimension showed a Cronbach’s alpha level of 0.76, wherein this study, the Cronbach’s alpha was 0.74.

#### Relational factors

In the ecological model, the variable “resident aggression” may be considered an individual factor of residents, but in this study, we measured aggressive acts directed towards staff, and thus, we included this variable as a relational factor. We measured resident aggression with a modified version of a scale (five items) developed and used by Malmedal et al. (2014) [22] in Norwegian nursing homes. We also used a modified version of a scale (four items) from Malmedal et al. (2014) [22] to measure care-related conflicts between nursing staff and residents. In both scales, the values were scored on a 4-point Likert scale ranging from ‘never’ to ‘more than once a week’. These two dimensions had not been excessively validated, but the authors reported acceptable Cronbach’s alpha levels of 0.79 on resident aggression and 0.77 on care-related conflicts. The study by Malmedal et al. (2014) [22] did, however, measure if nursing staff had *ever* experienced any acts of aggression/conflicts, while in the current study we wanted to measure the annual prevalence of such acts. Also, considering that resident aggression towards staff is highly prevalent, sometimes occurring daily [54], the scoring values were altered to a Likert scale ranging from 1 to 5; ‘daily, weekly, monthly, rarely, never’, where average scores were calculated for each scale; higher scores indicating less aggression/conflicts. In the current study, Cronbach’s alpha levels were 0.81 on resident aggression and 0.87 on care-related conflicts.

#### Institutional factors

In this study, we included three work environment factors and two facility features on the institutional level. Quantitative job demands were assessed by the General Nordic Questionnaire for Psychological and Social Factors at Work (QPS_Nordic_) [55], and we also measured staff’s experience of social interactions at work (support from nearest manager and support from co-workers) with subscales from the QPS_Nordic_ [55]. The QPS_Nordic_ is a widely used instrument specifically designed for the assessment of psychological, social, and organizational work conditions of employees from various sectors including the healthcare sector in Nordic countries [55]. The scale job demands contain four items, support from nearest manager contain three items, and support from co-workers contain two items, where all items are scored on a 5-point Likert scale ranging from ‘very seldom/never’ to ‘very often/always’, and average scores are calculated for each subscale [55]. In the job demand scale, higher scores indicate more demands, while in the other scales; higher scores indicate more support from managers and coworkers. In the validation study by Elo et al. (2000) [55], Cronbach’s alpha levels were 0.73 on job demands, 0.83 on support from manager, and 0.80 on support from co-workers, while in the current study, Cronbach’s alpha levels were 0.72, 0.85, and 0.73, respectively. We used a multilevel approach to explore the potential hierarchical interplay between individual and institutional factors with nursing staff nested within nursing homes. Thus, the median score of these three work environment scales was aggregated from the individual level to the nursing home level.

Facility size was measured by the number of beds. Statistics Norway’s centrality measure was used for the location of municipalities. This index reflects centrality based on peoples’ travel time to work and service functions, where the first level embrace the most central (biggest towns) and level six the least central municipalities (rural communities) [56]. In this study, these six levels were further categorized into three: urban (Levels 1–2), suburban (Levels 3–4), and rural (Levels 5–6).

### Ethical considerations

The Regional Committee for Medical and Health Research Ethics (May 2018, reference number: 2018/314) approved the study. The survey questionnaire did not include information concerning participants names or birth dates, and consent was obtained when the staff placed the questionnaire in sealed study containers. Participants were informed that they could not withdraw from the study after the questionnaires were placed in the sealed containers. Due to the data analyses, a unique code was assigned to each nursing home. Nursing staff were assured that this code was kept in a safe place and that no one could be identified in any reports or publications.

### Statistical analysis

Data were analysed with the software package Stata 16.1 [57]. We assessed normality with the Shapiro-Francia test, and no variables were normally distributed. The dependent variable was highly skewed towards ‘Never’; thus, the variable was dichotomized into ‘No abuse’ (never) and ‘Abuse’ (one or more incidents). Characteristics of individual, relational, and institutional factors are presented with percentages (frequencies) and median (range). Prevalence rates of psychological abuse, physical abuse, and neglect are described with percentages (frequencies). We used bivariate logistic regression to examine associations between the dependent variable and all independent variables identified in Table 1. Our choice of covariates to be included in the multivariate logistic regression model was guided by previous empirical investigations, knowledge of potential spurious factors, and/or a *p*-value < 0.2 [58, 59].

In logistic regression analyses, some basic assumptions must be met [59]. Firstly, the independent variables should be linearly related to the log odds of the dependent variable, which we tested with the ‘linktest’, and non-linear variables were improved with polynomial terms or dichotomised by the median score into equal groups. Secondly, the multivariate models should have little or no multicollinearity, which we tested with Spearman’s correlation coefficients ≥0.8, Tolerance (T) measures < 0.1, and Variance Inflation Factor (VIF) > 10 as indicators of multicollinearity [60]. Thirdly, there must be an adequate number [10–20] of observations per covariate to avoid an overfit model, which was not a problem in our large survey. Finally, logistic regression analyses require that observations be independent, but in this study, nursing staff were nested within nursing homes (clusters), and contextual effects (institutional factors) may have affected their responses. Consequently, we used multilevel mixed-effects logistic regression to test the variance between nursing homes, where the nursing staff was set at level 1 and nursing homes at level 2. Multilevel models ‘incorporate cluster-specific random parameters that account for the dependency of the data by partitioning the total individual variance into variation due to the clusters or higher-level units and the individual-level variation that remains’ (page 3258) [61]. We assessed the importance of these clusters with the intraclass correlation coefficient (ICC) and standard error (SE). Multilevel models correct for statistical dependence in the data by reducing the SE that otherwise may be considerably underestimated, and even with a low ICC-value, the best practice is not to ignore the clustering effect but to account for the effect using a multilevel approach [62, 63].

Effect sizes are presented as odds ratio (OR) with 95% confidence interval (CI) and exact *p*-values, and we will report results from the full models. The regression models’ overall fits to the data were assessed with the Hosmer-Lemeshow goodness-of-fit test table group [10], with a *p* > 0.05 indicating a well-fitted model. Missing data were removed. Our dependent variables had missing data ranging from 5.8–7.2%, but we chose not to replace missing values with the mean or median due to the highly skewed nature of the data [64]. Since we included many covariates, each with some missing data, we lost about 25% of observations in the full regression models. This may have caused our estimates to be less precise or biased if the complete cases differed systematically from the incomplete cases [65]. Considering that our remaining sample size was still large (*n* ≥ 2773), we chose not to compute multiple imputations of missing data. No design or post-stratification weights were added.

## Results

### Characteristics of nursing staff and nursing homes

Detailed descriptions of nursing homes and nursing staff are presented in Table 2. Nursing staff who responded were typically women (91.0%), with a median age of 41 years (range 16–75), where 42.1% were licensed practical nurses, and 65.9% had no continuous health education. Participating institutions ranged in size from eight to 161 beds (median 38.5), where 42% were located in suburban areas, 31% in urban, and 27% in rural areas.

**Table 2:** Characteristics of nursing staff (*N* = 3693) and nursing homes (*N* = 100)

Variables	Response values	n (%)^*^	Median (range)	*Missing, n (%)*
**Individual (staff)**				
Sex	Female	3362 (91.0)		*19 (0.5)*
	Male	312 (8.5)		
Age	Years		41 (16–75)	*236 (6.4)*
Professional occupation	Nursing assistant	1023 (27.7)		*47 (1.3)*
	Licensed practical nurse	1553 (42.1)		
	Registered nurse/social educator	1070 (28.9)		
Continuous health education	No	2433 (65.9)		
	Yes	1076 (29.1)		*184 (5.0)*
Overall health	Very good	1293 (35.0)		*21 (0.6)*
	Good	1923 (52.1)		
	Neither good nor bad	405 (11.0)		
	Bad	48 (1.3)		
	Very bad	3 (0.08)		
Exhaustion	No	2692 (72.9)		*40 (1.1)*
	Yes	961 (26.0)		
Psychological distress	No psychological distress	2939 (79.6)		*191 (5.2)*
	Psychological distress	563 (15.2)		
Quality of childhood	Very good	1814 (49.1)		*34 (0.9)*
	Good	1264 (34.2)		
	Average	386 (10.5)		
	Difficult	155 (4.2)		
	Very difficult	40 (1.1)		
Job satisfaction	Very satisfied	1659 (44.9)		*18 (0.5)*
	Satisfied	1583 (42.9)		
	Neither/nor	360 (9.7)		
	Unsatisfied	62 (1.7)		
	Very unsatisfied	11 (0.3)		
Intention to leave the job	No	2409 (65.2)		*64 (1.7)*
	Yes	1220 (33.0)		
Attitudes	Higher score = more positive attitudes^**^		28 (8–40)	*264 (7.2)*
**Relational**				
Resident aggression	Higher score = less aggression		4.2 (1–5)	*107 (2.9)*
	Dichotomised:^**, ***^			
	- High aggression (median 1.0–4.2)	1866 (50.5)		
	- Less aggression (median 4.3–5.0)	1720 (46.6)		
Care-related conflicts	Higher score = less conflicts		4.0 (1–5)	*129 (3.5)*
	Dichotomized:^**, ***^			
	- High conflicts (median 1.0–3.9)	1633 (44.2)		
	- Few conflicts (median 4.0–5.0)	1931 (52.3)		
**Institutional**				
Quantitative job demands	Higher score = more demands^***^		2.7 (1–5)	*0*
Support from manager	Higher score = more support^***, ****^		4.0 (1–5)	*0*
Support from co-workers	Higher score = more support^***, ****^		4.0 (1–5)	*0*
Facility size	Number of beds		38.5 (8–161)	*0*
Location of municipalities	Urban (levels 1–2)	31 (31.0)		
	Suburban (levels 3–4)	42 (42.0)		*0*
	Rural (levels 5–6)	27 (27.0)		

^*^due to rounding errors, not all numbers add up to 100%

^**^variable dichotomized due to non-linearity

^***^scale/score reversed in regression analysis

^****^median score aggregated from individual to nursing home level

### Risk factors of psychological abuse

The intraclass correlation coefficient of the psychological abuse model (intercept only) was 0.067, indicating that 6.7% of the variance of data was *between* nursing homes (Table 3). The ICC decreased to 4.7 and 3.7%, respectively, when individual and institutional factors were included in the models.

**Table 3:** Bivariate and multilevel mixed-effects logistic regression of risk factors of psychological abuse

Characteristics	Bivariate logistic regression			Mixed effect logistic regression model 1^*^			Mixed effect logistic regression model 2^*^		
	OR	95% CI	*p*	OR	95% CI	*p*	OR	95% CI	*p*
*Fixed effects*									
Nursing staff									
Sex _(0 = female, 1 = male)_	1.10	0.86–1.41	0.437	1.23	0.90–1.67	0.190	1.22	0.90–1.65	0.204
Age _(in years)_	1.00	0.99–1.00	0.598	1.00	0.99–1.00	0.468	1.00	0.99–1.00	0.366
Professional occupation _(ref: nursing assistant)_									
Licensed practical nurse	1.59	1.34–1.88	**< 0.001**	1.62	1.29–2.03	**< 0.001**	1.64	1.30–2.06	**< 0.001**
Registered nurse/social educator	1.68	1.39–2.01	**< 0.001**	1.74	1.37–2.21	**< 0.001**	1.77	1.40–2.25	**< 0.001**
Continuous health education _(0 = yes, 1 = no)_	0.95	0.81–1.10	0.494	–	–	–	–	–	–
Overall health _(1 = very good, 5 = very bad)_	1.31	1.18–1.44	**< 0.001**	1.10	0.96–1.25	0.176	1.09	0.96–1.25	0.195
Feeling exhausted _(0 = no, 1 = yes)_	1.73	1.48–2.02	**< 0.001**	0.95	0.77–1.18	0.640	0.94	0.76–1.16	0.554
Psychological distress _(0 = no, 1 = yes)_	1.96	1.62–2.37	**< 0.001**	1.45	1.14–1.85	**0.003**	1.46	1.14–1.86	**0.003**
Childhood _(1 = very good, 5 = very difficult)_	1.15	1.07–1.24	**< 0.001**	1.04	0.95–1.15	0.379	1.04	0.95–1.15	0.373
Job satisfaction _(1 = very satisfied, 5 = very unsatisfied)_	1.57	1.43–1.73	**< 0.001**	1.12	0.98–1.28	0.094	1.11	0.97–1.26	0.128
Intention to leave _(0 = no, 1 = yes)_	1.95	1.68–2.25	**< 0.001**	1.35	1.11–1.65	**0.003**	1.35	1.10–1.65	**0.003**
Attitudes _(8–40 ➔ higher score = poor attitudes)_	1.02	1.01–1.04	**< 0.001**	1.02	1.01–1.04	**0.012**	1.02	1.01–1.04	**0.012**
Relational									
Resident aggression _(0 = less aggression, 1 = high aggression)_	2.68	2.32–3.10	**< 0.001**	1.81	1.51–2.16	**< 0.001**	1.76	1.47–2.11	**< 0.001**
Care-related conflicts _(0 = few conflicts, 1 = high conflicts)_	2.76	2.39–3.18	**< 0.001**	2.31	1.95–1.75	**< 0.001**	2.33	1.96–2.77	**< 0.001**
Institutional									
Job demands _(1–5 ➔ higher score = more demands)_	1.62	1.19–2.21	**0.002**				0.89	0.50–1.58	0.700
Support from manager _(1–5 ➔ higher score = less support)_	1.64	1.34–2.00	**< 0.001**				1.56	1.08–2.25	**0.018**
Support from co-workers _(1–5 ➔ higher score = less support)_	1.75	1.38–2.21	**< 0.001**				1.23	0.80–1.90	0.352
Size _(number of beds)_	1.00	1.00–1.00	0.953				1.00	0.99–1.00	0.534
Location _(ref: urban)_									
Suburban	1.12	0.96–1.32	0.143				1.19	0.90–1.58	0.221
Rural	1.23	1.02–1.48	**0.032**				1.13	0.80–1.59	0.479
*Random effects*									
N				2777			2777		
Intraclass Correlation Coefficient (ICC)				0.047			0.037		
Standard Error (SE)				0.016			0.014		

Intercept only model: N (obs.) = 3427, N (groups) = 100, ICC = 0.067, SE = 0.016

^*^Model 1 = level 1-variables; Model 2 = level 1- and 2-variables

#### Adjusted psychological abuse model

As shown in Table 3, four individual staff factors, both relational factors, and one institutional factor made a statistically significant contribution to the psychological abuse model. Of the individual staff factors, predictors were 1) being a registered nurse/social educator (OR 1.77) or licensed practical nurses (OR 1.64), 2) reporting symptoms of psychological distress (OR 1.46), and 3) intention to leave the job (OR 1.35). Also, for every unit increase on the attitude scale (poor attitudes) (OR 1.02), nursing staff were more likely to perpetrate psychological abuse. Regarding relational factors, staff who reported high levels of resident aggression (OR 1.76) and conflicts with residents (OR 2.33) were more likely to perpetrate psychological abuse than staff who reported less aggression and fewer conflicts. Concerning institutional factors, the only predictor of psychological abuse was staff experiencing a lack of support from a manager (OR 1.56).

### Risk factors of physical abuse

The intraclass correlation coefficient of the physical abuse model (intercept only) was 0.027, indicating that 2.7% of the variance of data was *between* nursing homes (Table 4). The ICC decreased to zero when individual and institutional factors were included in the models.

**Table 4:** Bivariate and multilevel mixed-effects logistic regression of risk factors of physical abuse

Characteristics	Bivariate logistic regression			Mixed effect logistic regression model 1^*^			Mixed effect logistic regression model 2^*^		
	OR	95% CI	*p*	OR	95% CI	*p*	OR	95% CI	*p*
*Fixed effects*									
Nursing staff									
Sex _(0 = female, 1 = male)_	1.76	1.25–2.47	**0.001**	1.46	0.95–2.24	0.087	1.51	0.98–2.32	0.062
Age _(in years)_	1.00	0.99–1.01	0.910	1.00	0.99–1.01	0.705	1.00	0.99–1.01	0.690
Professional occupation _(ref: nursing assistant)_									
Licensed practical nurse	1.48	1.09–2.02	**0.012**	1.90	1.29–2.82	**0.001**	1.92	1.30–2.85	**0.001**
Registered nurse/social educator	1.98	1.45–2.71	**< 0.001**	2.48	1.67–3.68	**< 0.001**	2.49	1.68–3.70	**< 0.001**
Continuous health education _(0 = yes, 1 = no)_	1.03	0.80–1.33	0.795	–	–	–	–	–	–
Overall health _(1 = very good, 5 = very bad)_	1.27	1.08–1.49	**0.003**	1.02	0.83–1.25	0.858	1.02	0.83–1.25	0.878
Feeling exhausted _(0 = no, 1 = yes)_	1.59	1.25–2.02	**< 0.001**	1.00	0.73–1.37	0.995	1.00	0.73–1.38	0.987
Psychological distress _(0 = no, 1 = yes)_	2.01	1.54–2.62	**< 0.001**	1.61	1.15–2.24	**0.005**	1.62	1.16–2.27	**0.005**
Childhood _(1 = very good, 5 = very difficult)_	1.16	1.03–1.31	**0.013**	1.09	0.95–1.25	0.218	1.10	0.96–1.26	0.185
Job satisfaction _(1 = very satisfied, 5 = very unsatisfied)_	1.43	1.25–1.65	**< 0.001**	1.01	0.84–1.22	0.901	1.02	0.84–1.23	0.860
Intention to leave _(0 = no, 1 = yes)_	1.81	1.44–2.27	**< 0.001**	1.40	1.04–1.89	**0.026**	1.40	1.04–1.89	**0.028**
Attitudes _(8–40 ➔ higher score = poor attitudes)_	1.02	1.00–1.05	0.052	1.03	1.01–1.06	**0.014**	1.03	1.01–1.06	**0.013**
Relational									
Resident aggression _(0 = less aggression, 1 = high aggression)_	2.85	2.21–3.67	**< 0.001**	2.10	1.56–2.84	**< 0.001**	2.09	1.54–2.83	**< 0.001**
Care-related conflicts _(0 = few conflicts, 1 = high conflicts)_	2.81	2.20–3.59	**< 0.001**	2.18	1.64–2.89	**< 0.001**	2.18	1.64–2.89	**< 0.001**
Institutional									
Job demands _(1–5 ➔ higher score = more demands)_	1.48	0.89–2.46	0.133				1.35	0.66–2.75	0.409
Support from manager _(1–5 ➔ higher score = less support)_	0.97	0.70–1.35	0.877				0.65	0.41–1.04	0.072
Support from co-workers _(1–5 ➔ higher score = less support)_	1.35	0.91–1.98	0.134				1.20	0.70–2.05	0.518
Size _(number of beds)_	1.00	1.00–1.00	0.864				1.00	1.00–1.01	0.811
Location _(ref: urban)_									
Suburban	1.18	0.90–1.54	0.230				1.18	0.85–1.63	0.326
Rural	1.36	1.00–1.84	0.052				1.43	0.95–2.16	0.089
*Random effects*									
N				2797			2797		
Intraclass Correlation Coefficient (ICC)				9.90e-35			3.90e-35		
Standard Error (SE)				9.13e-19			4.75e-19		

Intercept only model: N (obs.) = 3477, N (groups) = 100, ICC = 0.027, SE = 0.020

^*^Model 1 = level 1-variables; Model 2 = level 1- and 2-variables

#### Adjusted physical abuse model

As shown in Table 4, four individual staff factors and both relational factors made a significant contribution to the physical abuse model. Staff predictors were 1) being a registered nurse/social educator (OR 2.49) or licensed practical nurse (OR 1.92), 2) reporting symptoms of psychological distress (OR 1.62), and 3) intention to leave the job (OR 1.40). The odds of physical abuse significantly increased with an OR of 1.03 for each unit increase on the attitude scale, indicating that poor attitudes were associated with perpetrating physical abuse. Regarding relational factors, staff who reported high levels of resident aggression (OR 2.09) and conflicts with residents (OR 2.18) were more likely to perpetrate physical abuse than staff who reported less aggression and fewer conflicts.

### Risk factors of neglect

The intraclass correlation coefficient of the neglect model was 0.020, indicating that 2.0% of the variance of data was *between* nursing homes (Table 5). The ICC decreased to 1.2 and 0.8%, respectively, when individual and institutional factors were included in the models.

**Table 5:** Bivariate and multilevel mixed-effects logistic regression of risk factors of neglect

Characteristics	Bivariate logistic regression			Mixed effect logistic regression model 1*			Mixed effect logistic regression model 2*		
	OR	95% CI	*p*	OR	95% CI	*p*	OR	95% CI	*p*
*Fixed effects*									
Nursing staff									
Sex _(0 = female, 1 = male)_	0.76	0.59–0.97	**0.026**	2.52	0.99–6.39	0.052	2.67	1.05–6.79	**0.039**
Age _(in years)_	1.00	1.00–1.01	0.408	1.00	1.00–1.01	0.235	1.00	0.99–1.01	0.227
Interaction age*sex	–	–	–	0.97	0.95–0.99	0.012	0.97	0.95–0.99	**0.009**
Professional occupation _(ref: nursing assistant)_									
Licensed practical nurse	1.73	1.46–2.04	**< 0.001**	1.75	1.41–2.19	**< 0.001**	1.77	1.42–2.21	**< 0.001**
Registered nurse/social educator	2.06	1.71–2.46	**< 0.001**	1.81	1.44–2.27	**< 0.001**	1.81	1.44–2.27	**< 0.001**
Continuous health education _(0 = yes, 1 = no)_	1.02	0.88–1.18	0.779	–	–	–	–	–	–
Overall health _(1 = very good, 5 = very bad)_	1.16	1.05–1.28	**0.003**	0.93	0.82–1.06	0.265	0.93	0.81–1.06	0.257
Feeling exhausted _(0 = no, 1 = yes)_	1.42	1.22–1.66	**< 0.001**	1.14	0.93–1.41	0.216	1.13	0.92–1.39	0.256
Psychological distress _(0 = no, 1 = yes)_	1.84	1.52–2.23	**< 0.001**	1.44	1.13–1.83	**0.003**	1.44	1.14–1.84	**0.003**
Childhood _(1 = very good, 5 = very difficult)_	1.16	1.08–1.25	**< 0.001**	1.13	1.03–1.25	**0.008**	1.14	1.03–1.25	**0.008**
Job satisfaction _(1 = very satisfied, 5 = very unsatisfied)_	1.44	1.31–1.58	**< 0.001**	1.13	0.99–1.28	0.064	1.13	0.99–1.28	0.069
Intention to leave _(0 = no, 1 = yes)_	1.83	1.59–2.12	**< 0.001**	1.40	1.16–1.71	**0.001**	1.39	1.15–1.69	**0.001**
Attitudes _(8–40 ➔ higher score = poor attitudes)_	0.96	0.95–0.97	**< 0.001**	1.15	1.03–1.28	**0.010**	1.15	1.03–1.28	**0.011**
Attitudes _(quadratic polynomial term)_	–	–	**–**	0.99	0.99–0.99	**0.001**	0.99	0.99–0.99	**0.001**
Relational									
Resident aggression _(0 = less aggression, 1 = high aggression)_	1.86	1.62–2.13	**< 0.001**	1.39	1.17–1.64	**< 0.001**	1.36	1.14–1.61	**0.001**
Care-related conflicts _(0 = few conflicts, 1 = high conflicts)_	2.02	1.76–2.32	**< 0.001**	1.96	1.66–2.33	**< 0.001**	1.97	1.66–2.33	**< 0.001**
Institutional									
Job demands _(1–5 ➔ higher score = more demands)_	1.65	1.21–2.23	**0.001**				1.56	0.99–2.48	0.057
Support from manager _(1–5 ➔ higher score = less support)_	1.24	1.02–1.50	**0.033**				0.93	0.69–1.26	0.655
Support from co-workers _(1–5 ➔ higher score = less support)_	1.27	1.01–1.59	**0.042**				0.99	0.70–1.41	0.966
Size _(number of beds)_	1.00	1.00–1.00	0.889				1.00	1.00–1.00	0.595
Location _(ref: urban)_									
Suburban	1.13	0.97–1.31	0.128				1.20	0.97–1.50	0.096
Rural	1.20	1.00–1.45	**0.049**				1.23	0.94–1.62	0.135
*Random effects*									
N				2773			2773		
Intraclass Correlation Coefficient (ICC)				0.012			0.008		
Standard Error (SE)				0.009			0.008		

Intercept only model: N (obs.) = 3460, N (groups) = 100, ICC = 0.020, SE = 0.009

^*^Model 1 = level 1-variables; Model 2 = level 1- and 2-variables

#### Adjusted neglect model

**Fig. 3:**
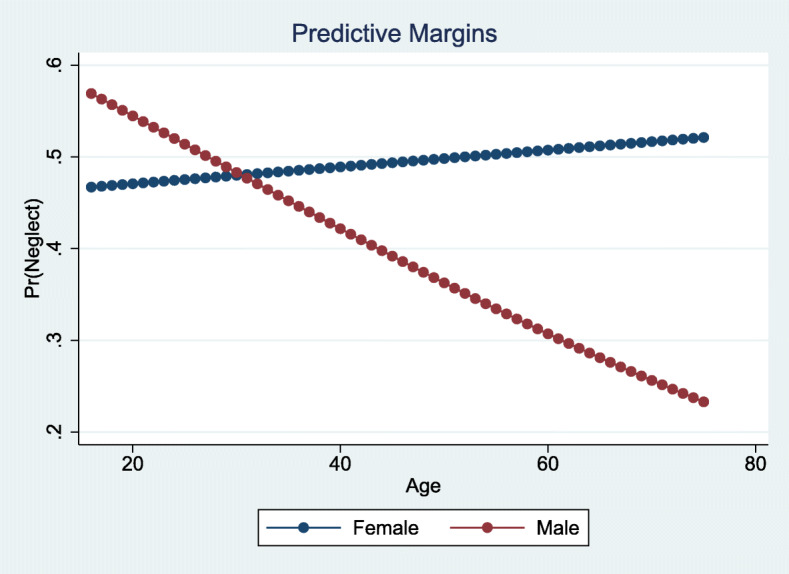
Margins plot of the interaction between gender, age, and neglect

As shown in Table 5, five individual staff factors and both relational factors made a significant contribution to the neglect model. Predictors of neglect were 1) being a registered nurse/social educator (OR 1.81) or licensed practical nurse (OR 1.77), 2) reporting symptoms of psychological distress (OR 1.44), 3) intention to leave the job (OR 1.39), and 4) poor quality of childhood (OR 1.14). Here, we found an interaction term between staff’s gender, age, and neglect, and by entering this interaction, the gender variable became significant. A margins plot illustrates that for each year, males reported fewer acts of neglect, whilst females reported more acts (Fig. 3).

**Fig. 4:**
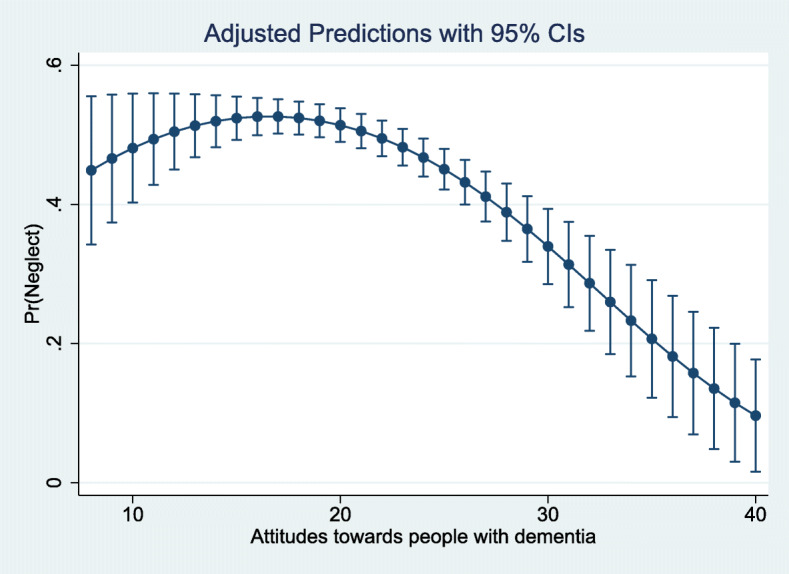
Margins plot of the quadratic polynomial term for attitudes and neglect

Further, our analyses showed that the variable ‘Attitudes’ had a curvilinear relationship with neglect, so by entering a quadratic polynomial term, a margins plot illustrates that staff with poor attitudes were more likely to perpetrate neglect to a certain point on the composite scale before they reported fewer acts of neglect (Fig. 4).

Concerning relational factors, staff who reported high levels of resident aggression (OR 1.36) and conflicts with residents (OR 1.97) were more likely to perpetrate neglect than staff who reported less aggression and fewer conflicts.

### Tests for statistical assumptions

All statistical assumptions were tested before entering multilevel modelling.

#### Linearity in the Logit

For the full models, the ‘linktest’ (hatsq) was not significant with *p* = 0.617 for the psychological model, *p* = 0.664 for the physical model, and *p* = 0.076 for the neglect model, indicating that all models were properly specified, and assumptions of linearity were met.

#### Multicollinearity

None of the three models had Spearman’s correlation coefficient ≥ 0.8, Tolerance value below 0.1, or VIF > 10, except for the quadratic polynomial term and interaction term in the neglect model.

#### Hosmer-Lemeshow test

The results from the Hosmer-Lemeshow test demonstrated a goodness-of-fit χ^2^ = 6.59 (*p* = 0.5814) for the psychological model, χ^2^ = 1.95 (*p* = 0.9824) for the physical model, and χ^2^ = 13.33 (*p* = 0.1010) for the neglect model, indicating that all models fit the data well.

## Discussion

This study of risk factors associated with staff-to-resident abuse in Norwegian nursing homes showed that various factors in the ecological model increase the likelihood of staff perpetrating psychological abuse, physical abuse, and neglect. The predictors most strongly found to be associated with all three types of abuse were 1) being a registered nurse/social educator or licensed practical nurse, 2) reporting symptoms of psychological distress, 3) considering leaving the job, 4) reporting poor attitudes towards persons with dementia, 5) and experiencing care-related conflicts and resident aggression. Other predictors were poor quality of childhood (neglect) and lack of support from a manager (psychological abuse).

### Individual staff factors

Concerning individual staff factors, the strongest predictor found associated with all three types of abuse was being a registered nurse/social educator or licensed practical nurse, compared to nursing assistants with no formal health education. This was also reported in a Norwegian nursing home study in 2014 [22], but it was inconsistent with other studies, suggesting that staff with lower education are more likely to perpetrate elder abuse [29, 30]. These opposite findings are not easily explained as many studies suggest that higher education and more knowledge are protective factors against elder abuse. Thus, a Cochrane review from 2016 [66] indicated ambiguity as to whether existing educational interventions lead to changes in staff behaviour and a reduction in elder abuse. One plausible explanation of our finding may be that health-educated nursing staff are more often allocated to work with agitated residents, hence experience and report more acts of abuse. Another explanation may be that health-educated staff with more training and knowledge of ethics and moral practice [67] reflect more critically upon their practices and how their behaviours affect residents and, hence, they more easily recognize and report acts of a negative character. Also, registered nurses/social educators and licensed practical nurses hold more permanent positions than temporary nursing assistants, who often work on an hourly basis, and this difference may explain our finding. For example, staff may consider acts of neglect, such as not giving appropriate oral care or ignoring a resident, as a systemic failure due to time restraints rather than their responsibility, and perhaps permanently employed staff are more prone to report such acts to make changes in the system. Furthermore, compared to staff working full or part-time, nursing assistants may not experience the same level of burnout, which is found to have a mediating role between different work environment factors and elder abuse [68]. Nevertheless, this inconsistency in education and knowledge related to elder abuse should be further explored in well-constructed and high-quality studies [66].

Another predictor found associated with all three types of abuse was nursing staff’s symptoms of psychological distress, which is consistent with a national study in Ireland that found poor mental health to be a predictor of staff-to-resident abuse [21]. Other studies have focused more on staff’s symptoms of burnout and emotional exhaustion and found these to be strong predictors of elder abuse [21, 23, 30, 32, 69]. We also measured the staff’s feelings of exhaustion, but no associations with perpetrating abuse were evident, which is surprising considering the reported strength of this factor. We speculate whether this inconsistency is because we measured exhaustion with one item only, where other studies have used more comprehensive burnout instruments such as the Maslach Burnout Inventory [21, 30, 69]. Vasconcelos et al. (2016) [70] conducted a review of nursing staff’s mental health and factors associated with the workplace and work process, and they found that high job demands, work pressure, violence and aggression, and poor relationships with the nursing team and managers exerted a negative impact on staff’s mental health.

Psychological problems stemming from work-related factors depend on staff’s personalities and experiences [70], and it is well documented that adverse childhood experiences are associated with an array of mental and physical health issues in later life [71]. Experiencing a poor-quality childhood may be related to psychological distress, but after controlling for other factors, we found that the staff’s poor childhood made a unique contribution as a predictor of neglect. To the best of our knowledge, this is not explored in other studies of staff-to-resident abuse. A recent study found an association between being a victim of child abuse and perpetrating elder abuse in adult life [25], but this intergenerational transmission of violence may not be directly attributable to formal caregivers perpetrating elder abuse in nursing homes. Shaw (1999) [72] found that staff in nursing homes who had been victims of domestic violence became sensitized to the invasion of personal space and reacted viscerally by committing physical abuse. We did, however, find that poor quality of childhood was associated with acts of neglect and not physical abuse, but one may assume that staff members’ early life stressors may manifest in a variety of ways that also may affect how they provide care to residents. Nevertheless, we do not fully understand the mechanism and causal effects of psychological distress, feelings of exhaustion, and adverse childhood experiences related to staff-to-resident abuse, and these predictors should receive more attention in future studies.

Job satisfaction has been recognized as one of the most persuasive factors influencing nursing staff’s intention to remain or quit the job [73]. Interestingly, we found that staff considering leaving their jobs was a predictor of perpetrating all types of abuse, but job satisfaction was not an associated factor. This is inconsistent with other studies that have found staff’s dissatisfaction as a predictor of staff-to-resident abuse [22, 23, 32]. Job satisfaction is defined as an emotional feeling influenced by several factors such as working conditions and social relations [74], and we speculate whether this inconsistency in findings is caused by the use of a single item, where others have used more wide-ranging instruments covering several dimension of job satisfaction [22]. Pillemer and Moore (1989) [27] used intention to quit one’s job as an indicator of nursing home staff’s dissatisfaction, but the intention to leave may also be the result of other factors. Tummers et al. (2013) [75] found that the most important reasons that nurses in long-term care intended to leave their organizations were related to negative working atmospheres and insufficient development and career opportunities.

Ageism is a profound problem potentially affecting all levels of the ecological model, individual, relational, institutional, and social. Three deleterious components can influence older people’s health: age discrimination (i.e., detrimental treatment of older adults), negative self-perceptions of ageing (i.e., beliefs held about one’s ageing), and negative age stereotypes (i.e., beliefs about older adults in general) [76]. When measuring nursing staff’s attitudes towards people with dementia, we found that staff showing poor attitudes were more likely to perpetrate all types of staff-to-resident abuse. However, when measuring neglect, we found a curvilinear relationship, where staff with both poor and good attitudes towards persons with dementia perpetrated neglect. To the best of our knowledge, this finding is not reported somewhere else and should be further explored. In U.S. nursing homes, Pillemer and Moore (1989) [27] found that staff who viewed residents as children were more likely to commit abuse. In interviews with German nursing home staff, Goergen et al. (2004) [23] found that staff expressed infantilizing attitudes and believed that residents should be treated with indulgence and their behaviour restricted and controlled. One may presume that geriatric training, as well as self-selection of those who choose to go into geriatrics, could reduce ageism and negative attitudes, and a study by Almogue et al. (2010) [77] suggested that employees in geriatric hospitals had better attitudes towards older persons than physicians and nurses in general hospitals. This was also reported by Kada et al. (2009) [53], where specialized trained nurses had significantly more positive beliefs than staff without this specialization. In our study, almost 30% of staff had continuing education in health care, but no significant association with perpetrated abuse was evident. One may discuss whether poor attitudes towards older people should be included as an individual level staff factor, an institutional (cultural) factor, or a broader societal factor affected by the community or country in which the institution is situated. The nursing staff bring their personal experiences and beliefs into nursing homes, but institutions, or even units within institutions, may comprise a culture where older people are marginalized and devalued, and abusive acts are tolerated and condoned [14]. Finally, we found a significant interaction term between staff’s age and gender and neglect, where younger males perpetrated more acts of neglect, but this considerably decreased with higher age. In contrast, younger female staff perpetrated fewer acts of neglect, but here, acts gradually increased with higher age. To our knowledge, this interaction between age and gender associated with neglect has not been previously reported. The literature does, however, suggest that both females and males of all ages are perpetrators of abuse [4, 22, 26–28]. One plausible explanation may be that compared to males, females obtain a higher responsibility and burden of care tasks at home when establishing their own families [78]. Nevertheless, this difference between men, women, and age-related to elder abuse is not easily explained and should be further explored.

### Relational factors

Concerning relational factors, we found care-related conflicts strongly associated with staff perpetrating all three types of abuse, and this is consistent with other studies of elder abuse in institutional care [21, 22, 69, 79]. Residents suffering from dementia may for many reasons refuse personal care, food, or medications, and they may become angry or agitated in a way that challenges nursing staff [80]. How staff cope in these situations may be affected by personal factors such as psychological distress or attitudes towards older people, but also by the level of geriatric training and institutional factors such as lack of time and resources [20, 81]. Again, we did not find that health education or continuous healthcare education was a protective factor against staff-to-resident abuse. Nursing home staff are at high risk of being exposed to aggression from residents with dementia or cognitive impairments, and consistent with previous literature [21, 23, 28, 32], we did find that resident aggression such as pinching, beating, or sexually harassing nursing staff was associated with perpetrating abuse, and one may assume that many of these incidents occurred in care situations and created conflicts. Since many residents display NPS such as agitation and aggressive behaviours, long-term caregivers should be trained to cope in these situations, and a recent promising study by Lichtwarck et al. (2019) [82, 83] found that a targeted intervention in nursing homes helped staff to cope with residents exhibiting NPS.

Also, NPS may contribute to incidents of resident-to-resident aggression in nursing homes, and Schiamberg et al. (2012) [84] found that RRA was a risk factor for staff-to-resident abuse. Moreover, relatives may also commit abusive acts towards nursing home residents, but to our knowledge, this has only been explored in two studies [85, 86], where one found relative-to-resident abuse to be more prevalent than staff-to-resident abuse [85]. Polyvictimization is a recently added term in the field of elder abuse, even though a significant number of studies have for many years documented the co-occurrence of multiple types of elder abuse by one or more perpetrators [87]. Polyvictimization may exacerbate negative outcomes more than any singular form of abuse [88], and more research is needed to improve its recognition and response [89].

### Institutional factors

There exist few studies of institutional risk factors related to elder abuse, and most evidence is gathered from policy and practice inquiries [14]. Individual staff and resident characteristics may be related to institutional maltreatment. For example, stressful or poor work environments may increase the risk of staff burnout, which may manifest as exhaustion, fatigue, stress, and/or dissatisfaction, which in turn may trigger staff-to-resident abuse [24, 28, 32]. In contrast, nursing homes providing a stable and positive work environment generate satisfied staff who provide good quality of care [90]. In our study, we found one institutional factor associated with psychological abuse: lack of support from a manager. In the Czech Republic, Buzgova and Ivanova (2011) [32] reported that nursing home staff who perpetrated abuse were more often dissatisfied with their work environments, did not feel inspired by their leaders, and regarded their work as demanding. In our study, we only measured three dimensions of the work environment, while there exist numerous factors including staffing and resources, job autonomy, leadership style, workplace conditions, procedures and routines, teamwork, and safety climate [55].

Despite the increase in elder abuse research, many healthcare professionals and institutional leaders display poor knowledge of what constitutes elder abuse, do not perceive elder abuse as a common or serious problem, and lack awareness of how to identify and report incidents of elder abuse [20, 91, 92]. A recent prospective, single-blinded, cluster-randomized, controlled trial evaluated the effectiveness of an intensive training program and found this to improve primary care nurses’ knowledge, attitudes, and confidence in intervening with elder abuse [93].

In 2009, Malmedal et al. [94] found that nine out of ten staff members admitted perpetrating inadequate care in Norwegian nursing homes. Still, in 2020 Myhre et al. [92] reported that Norwegian nursing home leaders considered staff-to-resident abuse ‘an unthinkable event’ and perceived nursing staff’s rough handling of residents as ‘mainly unintentional and something that could happen when caring for residents with aggression or those who resist care’. Nursing home leaders’ perception of elder abuse is essential to prevent or reduce staff-to-resident abuse as their understanding and attitudes may affect how nursing staff provide resident care [92], and we suggest that future studies explore a wider dimension of the work environment related to staff-to-resident abuse.

## Strengths and limitations

When recruiting nursing homes, more of the larger institutions rejected participation, which may have introduced selection bias. The nursing homes did not differ in location or ownership, but one may reflect whether these homes had more institutional problems than participating nursing homes. This study was based on nursing staffs’ reports, which may have introduced response bias due to social pressure to not reveal information concerning themselves and/or lack of self-awareness, and another limitation is that our survey instrument measuring the prevalence of abuse had not been thoroughly tested and validated. Also, the instrument by Kada et al. (2009) [53] measuring attitudes towards people with dementia had only been translated and not validated in a Norwegian context. Also, our modified version of the instrument by Malmedal et al. (2014) [22] had not undergone a thorough validation. Nevertheless, we achieved adequate Cronbach’s alpha levels on all scales. Due to the cross-sectional study design, we only provide associations and no causal inferences of staff-to-resident abuse. Finally, we used the WHO’s ecological model to guide our choice of risk factors, and we only included factors on three out of the four levels and no resident factors or relative-relations factors; thus, considering the complexity of elder abuse leads us to believe that other non-included factors may be related to staff-to-resident abuse in nursing homes.

The large sample size of 100 nursing homes and 3693 nursing staff may be considered a strength of this study. Also, this study is one of the largest staff surveys worldwide examining the prevalence and risk factors of elder abuse in nursing homes. The response rate of 60.1% may also be considered acceptable compared to some of the other studies in the area [21, 23, 95, 96]. Few studies have explored the hierarchical structure of nursing staff nested within nursing homes and staff-to-resident abuse with a multilevel approach, and the low ICC values on elder abuse prevalence when comparing nursing homes may suggest that the study population was representative of the target population. However, considering the methodological concerns with some of the measurement instruments used, and bearing in mind the inherent complexity of measuring the true prevalence of elder abuse in nursing homes, caution is needed when interpreting the factors found associated with elder abuse in the current study.

### Implications

Understanding the complexity of elder abuse and identifying predictors of staff-to-resident abuse may contribute to the reduction and prevention of abuse, and we believe this study provides evidence that may have some implications for education, nursing home care, and future research.

The responsibility of taking care of older people in nursing homes must not be taken lightly, and managers should take time to understand staff members’ strengths and limitations when it comes to their physical and mental health, as well as their attitudes towards older persons in general [81]. Managers should promote a positive and safe work environment with a high level of social and psychological support of staff and recognize that these are beneficial factors contributing to a high quality of care that may reduce staff-to-resident abuse [20, 90, 97, 98]. Optimal staff density in nursing homes is debated, as it may not only be a matter of quantity: a high percentage of qualified staff may be more likely to prevent elder abuse than a high proportion of staff without geriatric training [23, 97]. Moreover, managers should create a safe environment for nursing staff to discuss their failures and successes, as opposed to an inward-looking culture with a punishing ethos [20]. Nevertheless, they should be aware of how to report and handle both minor and serious acts of staff-to-resident abuse as they do occur [4].

Elder abuse awareness, knowledge, and training should be encouraged in both nursing homes and educational institutions. Our findings indicate that special attention should be paid to relational factors such as how to cope with residents exhibiting agitated or aggressive behaviours, but also to a general understanding of and attitude towards caring for people with dementia. A more person-centred approach that embraces older people’s values, preferences, and autonomy may prevent staff-to-resident abuse in nursing homes [99].

Finally, our findings support the evidence of the previous literature that has explored risk factors on different levels of the ecological model; elder abuse is a complex and multifaceted phenomenon. However, most studies have assessed these risk factors with cross-sectional designs that do not contribute to the understanding of the underlying mechanism or causes of abuse. Hence, future studies should explore potential risk factors with prospective or qualitative designs, and at the same time, provide more research on step three in WHO’s public health approach: design, implement, and evaluate preventive interventions with a multifaceted strategy.

## Conclusions

The findings of this study underline the importance of using a multifaceted strategy to identify risk factors for elder abuse in nursing homes as we found several predictors of staff-to-resident abuse on different levels of the ecological model. However, future studies should explore risk factors and the underlying mechanism in qualitative and prospective studies and design preventive interventions with a multifaceted strategy.

## Data Availability

The dataset generated and analysed during the current study is available from the corresponding author on reasonable request. The full survey questionnaire used in this study exists only in Norwegian language but may be provided upon request to the corresponding author.

## Abbreviations

WHO: World Health Organization
NPS: Neuropsychiatric symptoms
SCL: Hopkins Symptom Checklist
HUNT: Nord-Trøndelag Health Study
QPS_Nordic_: General Nordic Questionnaire for Psychological and Social Factors at Work
SE: Standard error
OR: Odds ratio
CI: Confidence interval
T: Tolerance
VIF: Variance inflation factor
ICC: Intraclass correlation coefficient
NTNU: Norwegian University of Science and Technology
